# FDTransformer: A firn density prediction framework combining a self-attention transformer network with firn densification physics

**DOI:** 10.1016/j.isci.2025.113869

**Published:** 2025-10-28

**Authors:** Xueyu Zhang, Lin Liu, Houjun Jiang, Zhicai Luo

**Affiliations:** 1 National Gravitation Laboratory, MOE Key Laboratory of Fundamental Physical Quantities Measurement, and School of Physics, Huazhong University of Science and Technology, Wuhan 430074, People's Republic of China; 2 Department of Geomatics Science and Engineering, School of Civil Engineering, Anhui Jianzhu University, Hefei 230601, People's Republic of China

**Keywords:** glacial landscapes, glaciochemistry, environmental science, environmental monitoring

## Abstract

Accurate firn density estimation is essential for assessing glacier mass balance. However, the accuracy of existing firn densification models (FDMs) is limited by an incomplete understanding of firn compaction dynamics, particularly as firn structure alters in warming conditions. This study proposes the firn density prediction transformer (FDTransformer), a deep learning framework that combines firn densification physics to improve density estimation. By employing the transformer network with sequential self-attention mechanisms, the FDTransformer learns a nonlinear mapping between physical firn parameters input and observed firn density, enabling physics-to-density sequence transformation. These physically constrained parameters are estimated by applying physics-based FDMs. Evaluated using *in situ* measurements from three Greenland sites (Dye-2, KAN_U, and Summit) with varying firn evolution patterns, the FDTransformer reduces mean absolute error by 30%, 42%, and 24%, respectively, compared to the physics-based FDMs. This study demonstrates that combining deep learning techniques with firn densification physics can improve firn density assessment.

## Highlights

• A novel method for estimating firn density
• This method performs well at three sites with varying complexities of densification
• This method reduces density simulation biases compared to traditional densification models

## Graphical abstract

**Figure:**
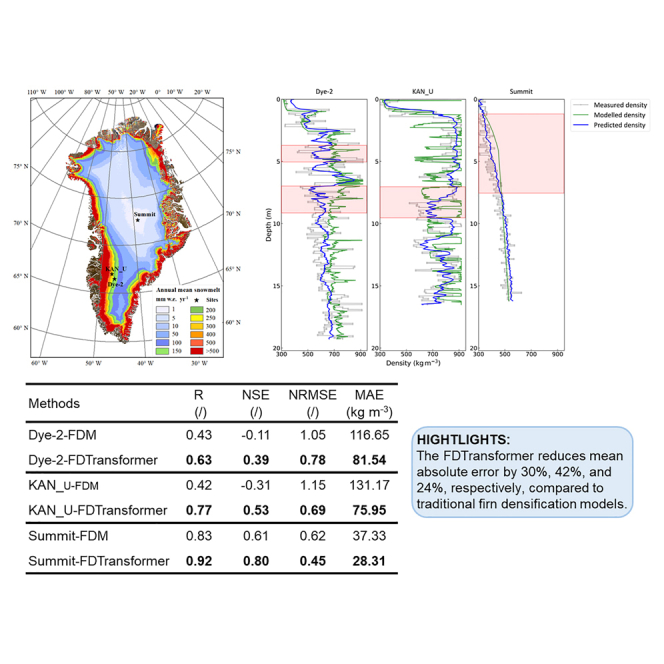

## Introduction

The accelerated loss of glacier mass is a key driver of global sea-level rise, highlighting the urgency of understanding glacier mass balance.^1^ A typical method to estimate glacier mass balance involves converting height (volume) changes measured from satellite altimetry into mass changes using firn density,^2^^,^^3^^,^^4^^,^^5^^,^^6^ which remains a primary uncertainty source in current mass balance assessment.^7^^,^^8^^,^^9^^,^^10^ Moreover, firn density and its spatial distribution are required to calculate the amount of air-filled pore space within the firn column (firn air content), which affects the meltwater retention potential and the capacity of firn to buffer surface meltwater into the ocean.^11^^,^^12^^,^^13^^,^^14^ Numerous studies have shown that firn density varies significantly in both space and time due to the varying surface climate conditions.^15^^,^^16^^,^^17^ Consequently, accurately estimating the firn density and its evolution under different climate conditions is essential.

Various methods have been developed to assess firn density. *In situ* measurements from firn cores and/or snow pits offer the most accurate understanding of firn density. However, these measurements can only provide snapshots of firn density in space and time due to the high cost of sampling and measurement, making them insufficient for comprehensive monitoring requirements.^18^ In early research, many empirical, semi-empirical, and physical firn densification models (FDMs) have been used to simulate firn density on both Antarctica and Greenland, owing to the absence of suitable techniques to observe the variations in firn density across the entire ice sheet.^4^ Empirical and semi-empirical FDMs establish relationships between densification rates and factors such as temperature and accumulation rate, with empirical parameters calibrated using observational density data.^2^^,^^3^^,^^4^^,^^19^^,^^20^^,^^21^ These models can simulate relatively accurate density profiles through calibration, which reduces uncertainties arising from incompletely understood firn densification processes. However, the observations used to calibrate empirical and semi-empirical firn models may not adequately represent future firn properties under a warming climate.^10^^,^^22^ Physical FDMs simulate densification using the material properties of snow and firn, based on constitutive relations between stress and strain.^23^^,^^24^^,^^25^ Although these physics-based models do not depend on observational tuning, they generally are built on strong assumptions and significant simplifications due to the incomplete understanding of the complex and dynamic interactions during densification. Under the context of global warming, the increased liquid water further complicates the physical mechanisms of densification, making it challenging for these physical models to fully capture firn dynamics.

Researchers have found sharply increased firn density and significant density variability within the upper firn column at sites experiencing snowmelt.^12^^,^^26^^,^^27^^,^^28^ As snowmelt increases, the agreement between observed and simulated firn density decreases,^2^^,^^4^^,^^10^^,^^22^ highlighting the limitations of current FDMs in the context of global warming. For example, Thompson et al.^10^ used two firn models to quantify firn properties across the Greenland Ice Sheet (GrIS) and found that the model bias is highest where liquid water is abundant. The primary challenge in modeling firn densification lies in the insufficient *in situ* data and the limited knowledge of the complex densification mechanisms. In recent years, data-driven machine learning and deep learning methods have attracted widespread attention for their ability to establish nonlinear mapping relationships between input and output.^29^^,^^30^^,^^31^ These methods are particularly suitable for addressing problems with complex mappings where accurate physical models are either unavailable or challenging to construct. For firn density assessment, Li et al.^32^ employed a random forest machine learning approach to capture the nonlinear relationship between firn density and satellite observations from microwave radiometers and scatterometers, thereby evaluating Antarctic dry firn density. However, their study was constrained by its reliance on satellite data, which limited the model’s ability to capture deeper stratigraphic variations and restricted it to focus on dry-snow zones due to wet firn substantially influencing microwave signals.

This study proposes a novel method for firn density estimation and evaluates its applicability across sites with varying firn evolution patterns. Specifically, we treat the simulation of firn density profiles as a sequence-to-sequence (S2S) conversion task and develop the firn density prediction transformer (FDTransformer), a framework for estimating firn density that combines a sequence self-attention deep learning model based on transformer architecture with firn densification physics. The transformer network is employed to autonomously learn the data-driven nonlinear mapping between physical firn parameters and observed firn density, enabling physics-to-density sequence transformation. These physically constrained firn parameters are derived from physics-based FDMs. Three sites, Dye-2, KAN_U, and Summit, representing various firn zones of the GrIS, are selected to validate the performance of the proposed FDTransformer model. At all three sites, the FDTransformer consistently reproduces bulk density trends and variability, achieving the highest accuracy at Summit, where firn density varies with depth in the simplest pattern. At Dye-2 and KAN_U, despite challenges posed by liquid water percolation and refreezing, the model captures key densification patterns, outperforming traditional FDMs, which struggle with meltwater-induced variations.^33^ Specifically, the FDTransformer achieves higher accuracy in firn density estimation, with mean absolute error (MAE) decreasing by 30%, 42%, and 24% at Dye-2, KAN_U, and Summit, respectively, compared to FDM values. The most substantial improvement occurs at KAN_U, which experiences the highest liquid water among the study sites, highlighting FDTransformer’s superior performance at sites experiencing abundant snowmelt. These results demonstrate that combining deep learning techniques with firn densification physics could improve firn density assessment, reducing uncertainties stemming from the limited understanding of densification mechanisms.

## Results

**Figure 1:**
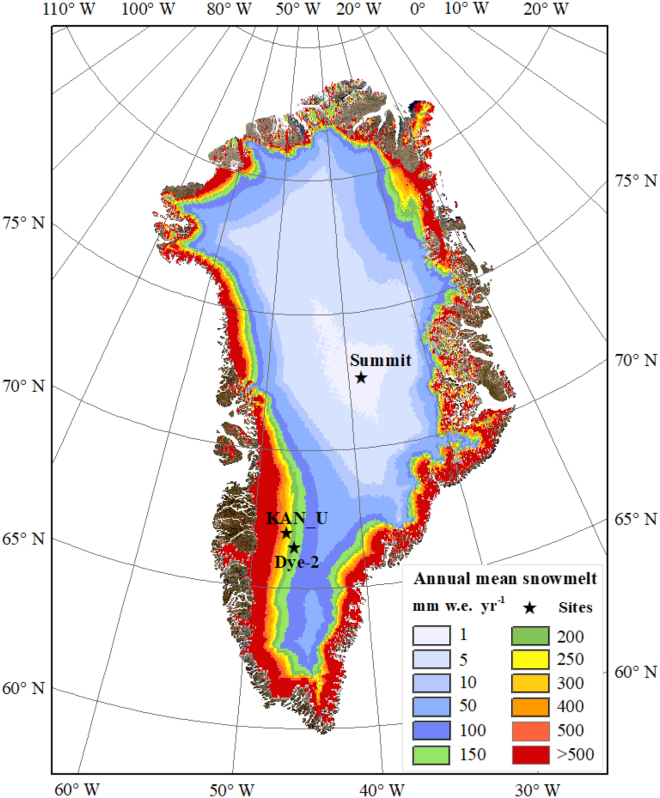
Geographic location of the three study sites on the GrIS Spatial distribution of the annual mean snowmelt rate across the GrIS (1958–2020) is calculated from RACMO2.3p2 (the Regional Atmospheric Climate Model version 2.3p2).

**Figure 2:**
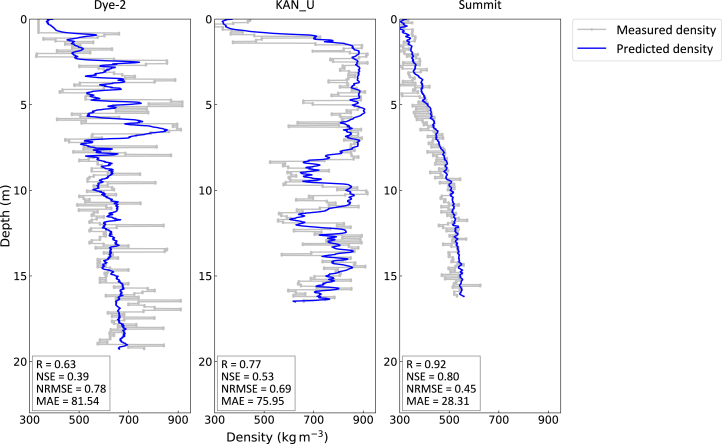
Comparison of the measured and predicted firn depth-density profiles at Dye-2, KAN_U, and Summit The measured density profiles and predicted density profiles are represented by gray dotted lines and blue solid lines, respectively. The correlation coefficient (R), NSE, NRMSE, and MAE are displayed in the bottom left of each panel.

To explore the effectiveness and generalizability of the proposed FDTransformer model in firn density estimation, we conducted case studies at Dye-2, KAN_U, and Summit, three sites of the GrIS. The geographical and climatic information of these sites is summarized in Table 1, and their spatial distribution is shown in Figure 1. These three selected sites exemplify typical firn zones of the GrIS: a site represents the percolation zone (Dye-2), a site represents the ice slab zone (KAN_U), and a site represents the dry-snow zone (Summit). The distinct climatic conditions at these sites govern distinct patterns of depth-dependent firn density evolution. The details of the *in situ* firn density profile data from the three sites used for model training, validation, and testing are provided in Table 2. Figure 2 presents the firn density profiles predicted by the FDTransformer on the test profiles, compared with the corresponding measured density profiles. The results demonstrate the model’s ability to simulate firn depth-density profiles at all three sites, despite the varying complexities of the underlying physical mechanisms. The FDTransformer successfully captures the general trends of firn density evolution and provides accurate predictions of firn density within reasonable uncertainty at each site.

**Table 1:** The geographical and climatic information of the three study sites

Site	Latitude (N)	Longitude (W)	Elevation (m a.s.l.)	Annual mean surface temperature (°C)	Annual mean accumulation (mm w.e.)	Annual mean snowmelt (mm w.e.)
Dye-2	66°28′33.20″	46°17′07.30″	2119	−18.34	426.32	145.79
KAN_U	66°59′59.57″	47°01′25.57″	1840	−16.79	463.38	282.83
Summit	72°34′38.93″	38°28′09.23″	3206	−28.40	202.34	0.52

The annual mean surface temperature, accumulation, and snowmelt are calculated based on RACMO2.3p2 for the period from 1958 to 2020. The latitude, longitude, and elevation for each site are the average position of all measured profiles at that site (see section in situ density profiles for details).

a.s.l., above sea level; w.e., water equivalent.

**Table 2:** *In situ* density profiles from firn cores

Profile	Core name	Date	Latitude (N)	Longitude (W)	Elevation (m a.s.l.)	Depth of core top (m)	Depth of core bottom (m)	Data source
D1	Dye-2-13-1	2013-05-05	66°28′39.29″	46°17′04.99″	2,119	0	16.64	1
D2	Dye-2-13-2	2013-05-05	66°28′21.36″	46°16′58.73″	2,119	0	16.45	1
D3	Dye-2-15	2015-05-21	66°28′39.76″	46°17′09.82″	2,126	0.81	19.27	2
D4	Dye-2-16	2016-05-06	66°28′21.36″	46°16′58.73″	2,126	0	17.37	2
D5	Dye-2-17-1	2017-05-11	66°28′21.36″	46°16′58.73″	2,126	0	22.97	2
D6	Dye-2-17-2	2017-05-13	66°28′40.94″	46°17′13.67″	2,112	0	26.68	3
D7	Dye-2-18	2018-05-09	66°28′40.80″	46°17′13.20″	2,112	0	19.78	3
D8	Dye-2-19	2019-05-19	66°28′40.80″	46°17′20.40″	2,113	0	20.97	3
K1	KAN_U-12-1	2012-05-01	67°00′00.90″	47°01′16.68″	1,840	0.80	10.68	1
K2	KAN_U-12-2	2012-05-01	67°00′00.90″	47°01′16.97″	1,840	0.59	10.49	1
K3	KAN_U-12-3	2012-05-01	66°59′53.70″	47°01′14.99″	1,840	0.17	10.26	1
K4	KAN_U-13-1	2013-04-27	67°00′00.90″	47°01′21.47″	1,840	0	19.12	1
K5	KAN_U-13-2	2013-04-28	66°59′54.13″	47°01′19.67″	1,840	0.79	15.94	1
K6	KAN_U-15	2015-05-05	67°00′01.51″	47°01′28.99″	1,840	0.65	14.40	4
K7	KAN_U-16-1	2016-04-26	67°00′01.37″	47°01′34.14″	1,838	0	7.97	5
K8	KAN_U-16-2	2016-04-28	67°00′01.37″	47°01′34.14″	1,838	0	16.51	4
K9	KAN_U-17	2017-04-28	67°00′00.90″	47°01′21.47″	1,840	0.78	23.27	4
K10	KAN_U-19	2019-05-10	66°59′59.90″	47°01′47.32″	1,840	0	4.92	3
S1	Summit-15-1	2015-05-28	72°34′38.71″	38°28′10.38″	3,203	0	15.79	5
S2	Summit-15-2	2015-05-28	72°34′38.60″	38°28′09.23″	3,205	0	7.68	5
S3	Summit-15-3	2015-05-28	72°34′38.78″	38°28′09.19″	3,203	0	15.93	5
S4	Summit-16	2016-05-16	72°34′39.29″	38°28′08.65″	3,209	0	16.20	5
S5	Summit-17	2017-05-20	72°34′39.29″	38°28′08.65″	3,209	0	22.17	5

Characteristics of the firn cores include core retrieval date, location, elevation, depth of core top and bottom below the surface, as well as data source. Data source refers to (1) Machguth et al.,^12^ (2) Vandecrux et al.,^42^ (3) Rennermalm et al.,^43^ (4) MacFerrin et al.,^27^ and (5) MacFerrin et al.^41^

At Dye-2, the FDTransformer exhibits a moderate correlation (R = 0.63) with measured firn density, suggesting some level of predictability, despite the complexity introduced by liquid water refreezing at certain depths. The model has the ability to capture the overall trend of firn density increasing with depth, which is primarily driven by mechanisms associated with dry firn compaction. The MAE of 81.54 kg m^−3^ indicates relatively minor deviations from measured values, and the normalized root-mean-square error (NRMSE) of 0.78 confirms that these deviations remain within the expected range of natural density variability at this site. Additionally, the FDTransformer can capture the sharp increases in firn density caused by liquid water refreezing at certain depths, particularly within the first 10 m of the firn column.

At KAN_U, the FDTransformer reproduces a few-meter-thick ice slab in the first few meters of the firn column, followed by a low-density zone and a subsequent high-density zone after the ice slab. The formation of ice slabs is a complex interaction among accumulation, densification, meltwater percolation, and refreezing, making their numerical simulation particularly challenging due to the tightly coupled nature of these interdependent mechanisms.^12^^,^^33^ By learning the site-specific firn density evolution pattern at KAN_U, the FDTransformer successfully reproduces ice slab formation and performs well in simulating this generally high firn depth-density profile (R = 0.77, Nash-Sutcliffe efficiency coefficient [NSE] = 0.53, NRMSE = 0.69, MAE = 75.95 kg m^−3^). The four improved metrics demonstrate the FDTransformer’s superior firn density prediction capability at KAN_U compared to Dye-2. This performance discrepancy likely stems from the KAN_U’s less variable density distribution, which enables the FDTransformer to capture the overall compaction trend more effectively.

At Summit, where liquid water is nearly absent, the FDTransformer achieves peak performance (R = 0.92, NSE = 0.80, NRMSE = 0.45, MAE = 28.31 kg m^−3^), demonstrating significantly better accuracy than at the melt-affected sites KAN_U and Dye-2. This enhanced accuracy may be attributed to Summit’s near-monotonic firn depth-density relationship, where density evolves predictably through steady compaction. The FDTransformer exhibits stronger learning capability for this simpler pattern, unaffected by dynamic meltwater percolation and refreezing processes that complicate modeling at Dye-2 and KAN_U.

## Discussion

### Transformer network with sequence self-attention enhances firn density assessment accuracy

**Figure 3:**
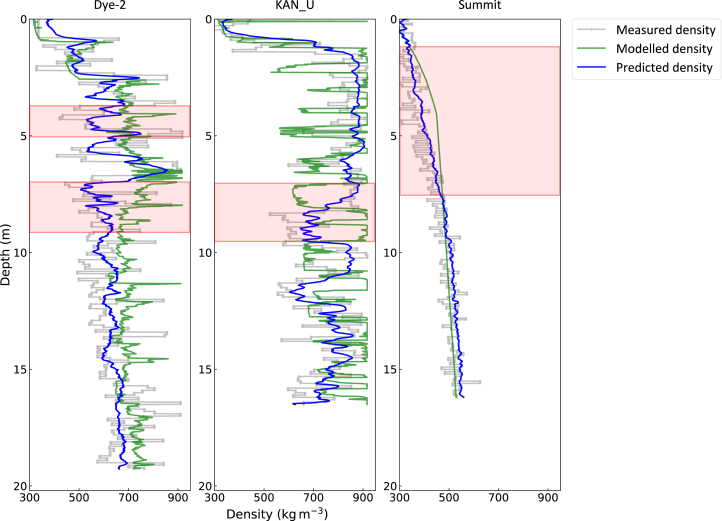
Comparison of measured, FDM-simulated, and FDTransformer-predicted firn depth-density profiles at Dye-2, KAN_U, and Summit Firn depth-density profiles measured from firn cores, simulated by the FDM, and predicted by the FDTransformer are represented by gray dashed line, green solid line, and blue solid line, respectively. The red rectangular box indicates the typical depth ranges where the density predicted by the FDTransformer is closer to the measured firn density than the density modeled by the FDM.

**Figure 4:**
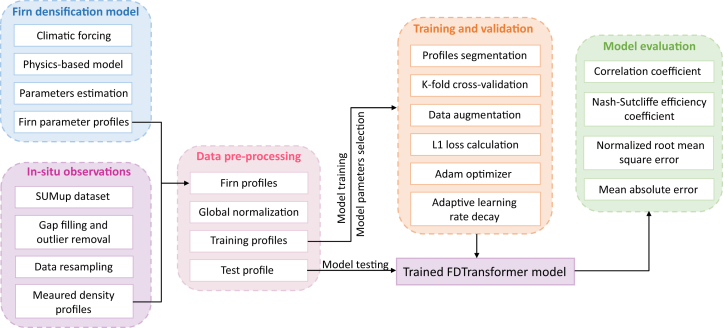
The framework of the proposed FDTransformer model for firn density estimation K represents the number of training profiles, which are used for the training and validation of the model.

**Figure 5:**
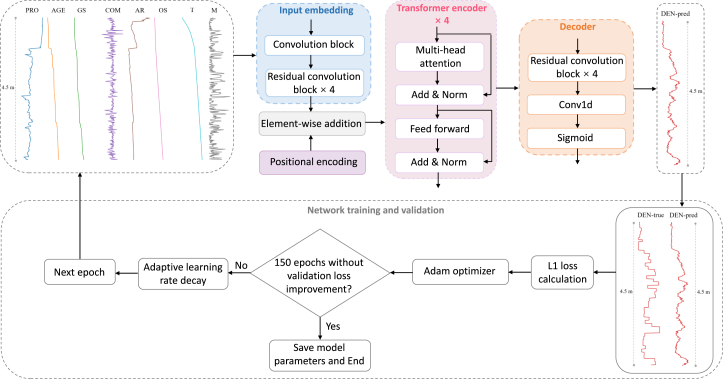
The architecture of the proposed FDTransformer model for firn density prediction within an epoch The input sequences include POR (porosity), AGE (age), GS (grain size), COM (compaction), AR (accumulation rate), OS (overburden stress), T (temperature), and M (mass). DEN-pred and DEN-true refer to the predicted density sequence and the measured density sequence, respectively. The Conv1d represents the 1D convolution layer with 64 kernels, a kernel size of 11, padding of 5, and a stride of 1. See also Figure S4.

In this section, we compare the performance of the FDTransformer with that of the FDM, using the predicted/simulated firn depth-density profiles on the test profiles at Dye-2, KAN_U, and Summit, respectively (Figure 3). It is important to note that the FDM mentioned here is the one used to generate the physical firn parameters (defined in Table 3, see physically constrained firn parameters section in STAR Methods for details). This comparison provides insight into how much the combination of deep learning techniques has improved the accuracy of firn density assessment. Both models are evaluated using the same metrics (Table 4). Firn deposition is a continuous, time-dependent process, with the depth-density profile reflecting layer-specific responses to deposition conditions. An overview of the proposed FDTransformer framework is presented in Figure 4, and its detailed network architecture within an epoch is depicted in Figure 5. Compared to the FDM, the self-attention mechanism in the FDTransformer considers the influence of other points in the sequence when predicting each depth point, which not only enhances prediction accuracy but also makes the trend of the predicted density curve closer to the actual density curve. Across all three sites, the FDTransformer demonstrates a clear ability to simulate firn depth-density profiles with high accuracy. The FDTransformer captures the broad trends and key features of firn density evolution, even with varying levels of complexity in the physical mechanism. The FDTransformer’s performance improves as the density profile becomes more predictable, as shown by the high accuracy at Summit. Even at Dye-2, where rapid changes in density due to liquid water refreezing introduce challenges, the FDTransformer still provides valuable insights into the general compaction trends and density variability.

**Table 3:** The meaning and units of the eight depth-dependent physical parameters generated by the densification model, as well as the variables and units of the climatic forcing used as inputs to the densification model

Variable name	Specific meaning (if applicable)	Unit
**Firn physical parameters**		

POR (porosity)	Porosity of each firn layer	N/A
AGE (age)	Age of the firn	years
GS (grain size)	The grain size of the firn, quantified by the squared grain radius	m^2^
COM (compaction)	Total compaction of each node since the previous time step	m
AR (accumulation rate)	The mean accumulation rate over the lifetime of each parcel of firn	m i.e., a^−1^
OS (overburden stress)	The overburden stress of each firn layer	kg m^2^ s^−2^
T (temperature)	Temperature of the firn	K
M (mass)	Mass of each firn layer	kg

**Climatic forcing data**		

Snowfall	–	mm w.e. day^−1^
Sublimation	–	mm w.e. day^−1^
Snowmelt	–	mm w.e. day^−1^
Total precipitation	Including snowfall and rainfall	mm w.e. day^−1^
Skin temperature	–	K
2-m air temperature	–	K

N/A, not applicable.

**Table 4:** Performance evaluation of FDM and FDTransformer in estimating firn depth-density profiles against measurements

Methods	R (/)	NSE (/)	NRMSE (/)	MAE (kg m^−3^)
Dye-2-FDM	0.43	−0.11	1.05	116.65
**Dye-2-FDTransformer**	**0.63**	**0.39**	**0.78**	**81.54**
KAN_U-FDM	0.42	−0.31	1.15	131.17
**KAN_U-FDTransformer**	**0.77**	**0.53**	**0.69**	**75.95**
Summit-FDM	0.83	0.61	0.62	37.33
**Summit-FDTransformer**	**0.92**	**0.80**	**0.45**	**28.31**

Evaluation metrics include the R, NSE, NRMSE, and MAE.

Values in bold represent better performance metrics.

At Dye-2, the FDTransformer outperforms the FDM in capturing the general trend of the firn density profile. As shown in Figure 3, particularly in the red rectangular box highlighted in the figure, the FDTransformer captures the density fluctuations and the overall trend more effectively than FDM, which struggles with these variations and overestimates firn density. The correlation coefficient for the FDTransformer (R = 0.63) is significantly higher than that of the FDM (R = 0.43), indicating a better fit to the measured density. Similarly, the NSE for FDTransformer (0.39) is much higher than that for FDM (−0.11), suggesting that FDTransformer better captures the overall variability in the density profile. The MAE for the FDTransformer (81.54 kg m^−3^) is 30% lower than that of the FDM (116.65 kg m^−3^), demonstrating its superior prediction accuracy. Despite the challenges posed by the complex density fluctuations due to liquid water refreezing, the FDTransformer demonstrates a clear advantage over FDM in simulating the overall density trend and density variability at Dye-2, with an NRMSE value of less than 1.

At KAN_U, where liquid water plays a dominant role in firn densification, the FDTransformer similarly performs better than the FDM. The red rectangular box in Figure 3 highlights areas where FDTransformer’s predictions are more accurate than FDM, effectively capturing the low-variability density profile. The correlation coefficient for FDTransformer (0.77) is substantially higher than that for FDM (0.42), and the NSE for FDTransformer (0.53) also exceeds that of FDM (−0.31). These improvements suggest that FDTransformer is more effective in capturing the relatively stable, high-density firn profile than FDM, influenced by the dominance of refreezing mechanisms in the densification process. FDTransformer reduces the MAE by 42% compared to FDM, highlighting FDTransformer’s superior performance at sites experiencing abundant snowmelt.

At Summit, where the firn density follows nearly a monotonically increasing trend with depth, both models perform well. The FDTransformer, as indicated by the red rectangular box in Figure 3, still provides superior predictions. The correlation coefficient for FDTransformer (0.92) is higher than that for FDM (0.83), and the NSE for FDTransformer (0.80) is significantly better than that for FDM (0.61), indicating that FDTransformer reproduces the firn density profile more accurately. Additionally, FDTransformer achieves an MAE of 28.31 kg m^−3^, which is 24% lower than that of FDM (37.33 kg m^−3^), reflecting its higher accuracy in simulating this nearly monotonically increasing firn density evolution pattern.

The FDTransformer’s combination of a sequence self-attention transformer network effectively reduces the biases in firn density estimation inherent in the conventional FDM, which could be attributed to three key factors. First, its attention-based transformer architecture excels at capturing long-range dependencies and subtle variations in sequential data, enabling more accurate and realistic density predictions, as evidenced by the significant reductions in MAE across all three sites. Second, the FDTransformer uses depth-related physical parameters generated by traditional FDMs as input while learning from actual measured density profiles. In this way, the FDTransformer combines physical insights from traditional FDMs with the actual observed data, enabling it to dynamically adjust and capture subtler variations in firn density that traditional models may overlook. Third, the FDTransformer learns the complex mapping relationship between physical parameters and the corresponding measured density, rather than relying solely on physical assumptions and predefined parameters. This enables the FDTransformer to optimize its model parameters based on actual measurements. This study demonstrates that combining deep learning techniques with firn densification physics significantly improves firn density estimation, achieving higher accuracy compared to traditional modeling approaches. Future work will focus on expanding the density dataset and optimizing the FDTransformer for broader applicability.

### Criteria for sequence length selection

**Figure 6:**
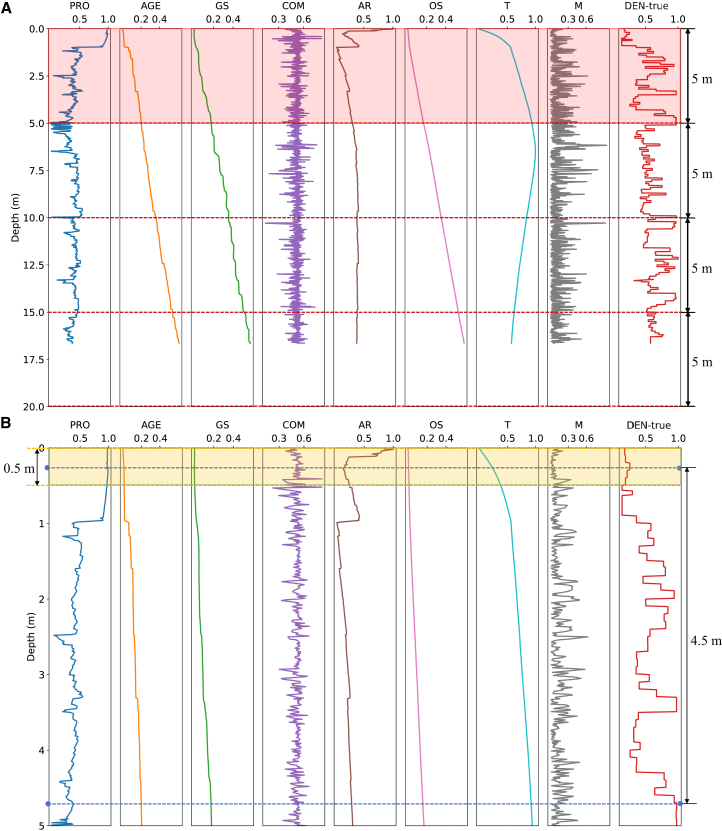
Preparation of globally normalized training and validation sequences, using profile D1 as an example (A) Schematic of profile segmentation. Profile D1 is divided into four 5-m-long sequences, with red dashed lines indicating the segment boundaries. (B) Schematic of the data augmentation strategy based on random starting point cropping, using the 5-m-long sequence inside the red rectangular box in (A) as an example. The yellow rectangular box shows the regions where the random starting point is located. The blue dashed line indicates a schematic of a 4.5-m-long sequence that can be used for model training and validation.

**Figure 7:**
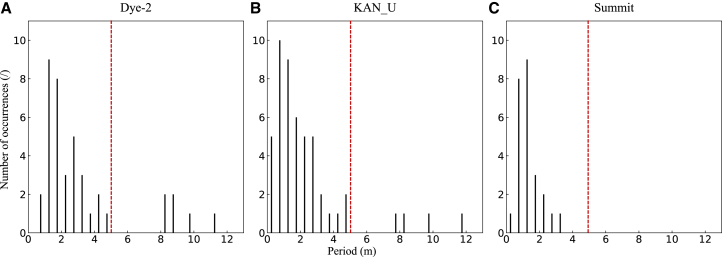
Criteria for sequence length selection Number of occurrences of the periods corresponding to the top five principal frequencies in each profile within different period intervals, with each interval width being 0.5 m, for multiple profiles at Dye-2 (A), KAN_U (B), and Summit (C). The black solid line represents the center of the period bins, and the red dashed line indicates a period of 5 m.

The selection of sequence length is crucial for determining the performance of the FDTransformer model, particularly when considering the periodicity and underlying patterns within the firn density profiles. In this study, the density profiles from Dye-2, KAN_U, and Summit are segmented into 5-m-long sequences for model training and validation (Figure 6, see STAR Methods for details), which is guided by the analysis of periodic components in the profiles of each site. We calculate the periods associated with the first five principal frequencies of each profile through the fast Fourier transform and then statistically analyze the number of occurrences of these periods across different period intervals, with each interval being 0.5 m (Figure 7).

As illustrated in Figure 7, all three sites exhibit a concentrated period distribution within the 0–5 m range, indicating that 5-m-long sequences are representative of the overall profiles. In addition, the choice of a 5-m sequence length strikes a balance between capturing sufficient detail and maintaining computational efficiency. On the one hand, shorter sequences would result in an overly fragmented representation of the density profile, potentially leading to a loss of contextual information. On the other hand, longer sequences could introduce unnecessary complexity without substantially improving the model’s ability to capture critical information, as most of the meaningful variations in the profiles are concentrated within the 5-m scale. The 5-m-long sequences, therefore, represent an optimal compromise, allowing the model to effectively capture key features in the firn depth-density profiles, while avoiding irrelevant noise or excessive detail. The improved accuracy of firn density assessment across all three sites further demonstrates the suitability and robustness of this sequence length. Despite differing physical conditions, such as varying liquid water content and firn densification mechanisms, the 5-m-long sequences allow the model to learn the primary patterns driving firn density change at each site.

### Limitations of the study

Extensive deep learning studies have demonstrated that the quantity and quality of samples are key factors influencing model performance,^34^^,^^35^ a conclusion that has been widely validated across different model architectures. Our study further verifies this perspective through comparative analysis of model performance at shallow (0–10 m) and deep (10 m to the maximum depth of the core) layers. As shown in Table 5, at the three study sites (Dye-2, KAN_U, and Summit), the average sample numbers of training profiles in shallow layers (1,000, 921, and 523, respectively) are significantly larger than those in deep layers (861, 343, and 543). This quantitative advantage enabled the model to learn more comprehensive feature representations from shallow-layer data. Moreover, the quality of deep core samples is often compromised by drilling challenges, introducing anomalies like the low-density feature at around 18 m in profile D1 (Figure S1), which persist despite preprocessing efforts. Although the FDTransformer’s self-attention mechanism demonstrates strong representational capacity, its sensitivity to such noise and outliers constrains robust feature extraction from deep firn data. This qualitative advantage enables the FDTransformer to learn more reliable representations of shallow-layer features. Consequently, the FDTransformer demonstrates consistently superior overall performance in shallow layers across all three sites, as supported by the three evaluation metrics (R, NSE, and NRMSE). For example, at Dye-2, the model exhibits markedly better performance (R = 0.67, NSE = 0.45, NRMSE = 0.74) in shallow layers compared to deep layers (R = 0.33, NSE = 0.11, NRMSE = 0.95).

**Table 5:** Performance evaluation of FDTransformer in estimating firn depth-density profiles against measurements for shallow (0–10 m) and deep (10 m to the maximum depth) firn layers

Site name	Depth range	Sample number	R (/)	NSE (/)	NRMSE (/)	MAE (kg m^−3^)	CV (%)
Dye-2	0–10 m (shallow)	1000	**0.67**	**0.45**	**0.74**	92.40	27.02
Dye-2	>10 m (deep)	861	0.33	0.11	0.95	**69.81**	**14.66**
KAN_U	0–10 m (shallow)	921	**0.84**	**0.63**	**0.61**	**72.20**	22.42
KAN_U	>10 m (deep)	343	0.48	0.16	0.92	81.70	**14.19**
Summit	0–10 m (shallow)	942	**0.86**	**0.67**	**0.58**	30.18	16.33
Summit	>10 m (deep)	543	0.41	−0.19	1.09	**25.31**	**5.71**

Sample number represents the average number of samples per depth range in the training profiles. CV quantifies relative variability of measured firn density, calculated as the ratio of the SD to the mean. Values in bold represent better performance metrics and lower relative variability.

CV, coefficient of variation.

The depth-dependent variations in model performance may also be related to the differential complexity of densification processes across firn layers. At Dye-2 and Summit, shallow layers exhibited higher MAEs compared to the deep layers despite the advantage in data quantity and quality, which correlates strongly with greater variability in near-surface firn density. Specifically, at Dye-2 and Summit, the coefficient of variation in shallow layers (27.02% and 16.33%, respectively) consistently exceeded that in deep layers (14.66% and 5.71%, respectively). This increased variability probably stems from the shallow firn’s heightened sensitivity to surface climate conditions such as temperature fluctuations, wind redistribution events, and radiation balance. Notably, while MAEs are slightly higher in shallow layers, their superior NRMSE values indicate better overall prediction reliability in near-surface firn layers. Results from KAN_U further demonstrate the importance of sample quantity. Despite higher variability in shallow layers, the FDTransformer achieves a lower MAE (72.20 kg m^−3^ compared to 81.70 kg m^−3^ in deep layers) when the sample quantity is approximately 3-fold, indicating that sufficient data volume can partially offset modeling challenges posed by high variability. These findings suggest that future studies should integrate deep learning techniques with more high-quality *in situ* measurements, particularly at sites experiencing frequent melt-refreeze cycles and complex firn densification processes, to better characterize vertical firn heterogeneity and improve model performance.

## Resource availability

### Lead contact

Requests for further information and resources should be directed to and will be fulfilled by the lead contact, Lin Liu (liulin616@hust.edu.cn).

### Materials availability

This study did not involve materials.

### Data and code availability

• The original training datasets, FDTransformer model outputs, and analysis code supporting this study are available at Figshare: https://doi.org/10.6084/m9.figshare.30052993^36^ with CC-BY 4.0 license.
• The RACMO2.3p2 data are available from Brice Noël without conditions.^37^
• The Community Firn Model code is publicly available under the MIT license at GitHub: https://github.com/UWGlaciology/CommunityFirnModel.^38^
• The depth-density profiles of total 23 cores used in this study can be found at Arctic Data Center: https://doi.org/10.18739/A2M61BR5M^39^ and https://doi.org/10.18739/A2CZ3263B,^40^ respectively.
• Any additional information required to reanalyze the data reported in this paper is available from the lead contact upon request.

## Acknowledgments

This research was financially supported by the 10.13039/501100001809National Natural Science Foundation of China (grant nos. 42274028 and 41704023) and the Doctoral Research Foundation of 10.13039/501100015673Anhui Jianzhu University (no. 2021QDZ07). We acknowledge Christopher Max Stevens for the open-source CFM model and his assistance in modeling the firn densification model. We would also like to thank Baptiste Vandecrux, Jing Xiao, and the members of the Geological Survey of Denmark and Greenland for compiling the available core data.

## Author contributions

Conceptualization, X.Z. and L.L.; methodology, X.Z. and H.J.; investigation, X.Z., L.L., H.J., and Z.L.; writing – original draft, X.Z. and L.L.; writing – review & editing, X.Z. and H.J.; funding acquisition, L.L. and H.J.; resources, L.L. and Z.L.; supervision, L.L., H.J., and Z.L.

## Declaration of interests

The authors declare no competing interests.

## STAR★Methods

### Key resources table

**Table:** 

REAGENT or RESOURCE	SOURCE	IDENTIFIER
**Deposited data**		

Original datasets for model training, validation, and testing	This paper	https://doi.org/10.6084/m9.figshare.30052993
FDTransformer model outputs	This paper	https://doi.org/10.6084/m9.figshare.30052993
Analysis code of model outputs	This paper	https://doi.org/10.6084/m9.figshare.30052993
RACMO2.3p2 data	Noël et al.^37^	N/A
Community Firn Model (CFM)	Stevens et al.^38^	https://github.com/UWGlaciology/CommunityFirnModel
Original depth-density profiles data	Vandecrux et al.^39^	https://doi.org/10.18739/A2M61BR5M
Winter snow density	Xiao et al.^40^	https://doi.org/10.18739/A2CZ3263B

**Software and algorithms**		

Python	https://www.python.org/	3.10.7
Pycharm	https://www.jetbrains.com/pycharm/	2022.2.2
ArcGIS	https://www.esri.com/	10.8
Microsoft PowerPoint	https://www.microsoft.com/zh-cn/microsoft-365/powerpoint	N/A
Microsoft Word	https://www.microsoft.com/zh-cn/microsoft-365/word	N/A
Microsoft Excel	https://www.microsoft.com/zh-cn/microsoft-365/excel	N/A

### Experimental model and study participant details

#### Study sites

To explore the performance of the proposed FDTransformer model in firn density prediction, case studies are conducted at Dye-2, KAN_U, and Summit, three typical sites of the GrIS. The characteristics and geographical information of the three sites are shown in Figure 1 and summarized in Table 1. Dye-2 (66°28′33.20″ N, 46°17′07.30″ W, 2119 m a.s.l.) is a typical site within the percolation zone, where snowmelt occurs on the surface every summer. The meltwater subsequently percolates into the snow and firn, releasing latent heat as it refreezes into ice lenses and/or ice layers. High snow accumulation and moderate snowmelt make densification at Dye-2 primarily driven by overburden stress. Meanwhile, the refreezing of liquid water significantly increases the density at certain depths, resulting in considerable density variability at this site. Located near the equilibrium line at a relatively low elevation, KAN_U (66°59′59.57″ N, 47°01′25.57″ W, 1840 a.s.l.) exhibits an annual mean snowmelt of 282.83 mm w.e. (water equivalent) from 1958 to 2020, nearly double that of Dye-2 (145.97 mm w.e.) during the same period. The substantial snowmelt at KAN_U makes it a representative site within the ice slab zone, where ice lenses and/or ice layers accumulate to form thick ice slabs, thereby impeding deep percolation of meltwater.^41^ At KAN_U, firn densification is predominantly driven by the refreezing of liquid water, leading to generally high firn densities. Summit (72°34′38.93″ N, 38°28′09.23″ W, 3206 a.s.l.), a representative site in the dry-snow zone, is characterized by almost negligible snowmelt events and consistently low temperature. At this site, firn densification is mainly driven by overburden stress, with firn density generally increasing with depth and exhibiting minimal density variability. These three sites are thus typical for assessing the capability of a model to estimate firn density under distinct firn evolution patterns.

#### *In situ* density profiles

Field-measured firn density profiles are applied as the target sequences for the FDTransformer, with 8, 10, and 5 profiles collected at Dye-2, KAN_U, and Summit, respectively (Figures S1–S3). These profiles were derived from punctual *in situ* measurements of firn cores and compiled in the Surface Mass Balance and Snow on Sea Ice Working Group (SUMup) dataset.^39^ Each firn core was cut into shorter segments, and the density of each core segment was calculated using the segment’s length, weight, diameter, and volumetric intactness data.^28^ Detailed characteristics of these firn cores are summarized in Table 2. The naming convention for the cores follows the standard established by Rennermalm et al.,^28^ where each core is labelled with the site name, drilling year, and a sequential number if multiple cores were drilled in the same year (e.g., Dye-2-13-1). Most of the studied cores (20 out of 23) exceeded 10 m in length, with 5 cores extending beyond 20 m, which is well beyond the pore close-off depth where firn transitions into ice at Dye-2 and KAN_U.^28^ To ensure consistency across the dataset, only the upper 20 m of the longer cores are used for training and validation, while the shorter cores are used in their entirety. This approach focuses on the depth range covered by most cores, avoiding potential biases introduced by including greater depths. Furthermore, the upper 20 m of firn shows the strongest response to liquid water percolation, making this depth range sufficient for studying firn evolution under the forcing of liquid water.

If density data for a specific core segment are missing, the mean density of the two adjacent core segments is assigned to fill the gap. Firn densities that are anomalously low (less than 80% of the values predicted by the Herron-Langway model; Rennermalm et al.^28^) are replaced with the average density of the nearest core segments above and below. Density values exceeding 917 kg m^-^^3^ (the density of pure ice) are considered unreasonably high (Rennermalm et al.^28^) and adjusted to 917 kg m^-^^3^. Moreover, for firn cores with a non-zero initial depth (e.g., Dye-2-15, KAN_U-12-1, KAN_U-12-2, KAN_U-12-3, KAN_U-13-2, KAN_U-15, and KAN_U-17), the firn densities prior to the initial depth are assumed to be the same as those of winter snow, which had been deposited on the surface since the end of the previous melting season.^44^ Due to differences in depth and resolution across the density profiles, the original density data are unsuitable for direct input into the FDTransformer model during training, validation, and testing. To ensure consistency and meet the input requirements of the model, the firn density data are resampled to a uniform resolution of 0.01 m.

#### Physically constrained firn parameters

In this study, eight depth-dependent firn parameter sequences (Table 3), generated by the firn densification model, serve as input sequences for the FDTransformer. These physically constrained parameters, including POR (porosity), AGE (age), GS (grain size), COM (compaction), AR (accumulation rate), OS (overburden stress), T (temperature), and M (mass), are key physical quantities related to firn density. Each parameter sequence represents a specific firn property and implicitly contains valuable geologic information, such as grain size, firn temperature, as well as the degree of densification. Here, we use the Community Firn Model (CFM),^45^ designed to simulate physical processes in firn, as the modelling framework to simulate these physical parameters. The modular nature of the CFM model allows users to freely choose specific modules and parameterizations they would like to implement for a given model run. For the firn parameter estimation, we use modules for firn density and temperature evolution, grain size evolution, meltwater percolation and refreezing, variable surface density, and sublimation. Multiple dry-firn densification models are contained in the CFM. Here, we use the stress-based dry-firn densification scheme (represented as CROCUS in CFM) across all three sites. The CFM model includes liquid water schemes of varying complexities, yet recent work by Verjans et al.^26^ suggests no evidence that more complex schemes yield better performance.^4^ Considering the differences in liquid water flux across the three sites, we apply the “tipping-bucket” scheme at Dye-2, the Darcy-flow scheme at KAN_U, and the non-percolation scheme at Summit. After the CFM model runs, the firn layers are interpolated to generate a depth sequence at 0.01 m intervals, and the physical parameters are interpolated to obtain their corresponding values at each depth in the sequence.

The climatic forcing data (Table 3) used in this study for the densification model are derived from RACMO2.3p2 (the Regional Atmospheric Climate Model version 2.3p2), which has been demonstrated to significantly improve firn densification modelling.^22^ RACMO2.3p2 is forced at the lateral boundaries by a combination of European Centre for Medium-Range Weather Forecasts (ECMWF) reanalysis datasets from ERA-40 over 1958-1978, ERA-Interim over 1979-1989, and ERA5 over 1990-2020.^37^ The data of snowfall, sublimation, snowmelt, total precipitation, and 2 m air temperature are statistically downscaled on a daily basis from RACMO2.3p2 at 5.5 km resolution onto a 1 km grid for the period 1958-2020. In addition, skin temperature is available on a daily basis for the same time period but on the native RACMO2.3p2 grid at 5.5 km. To ensure that no unnecessary transients are introduced during the simulation, a sufficiently long spin-up interval is applied to generate a firn column in dynamic equilibrium with the climate. Here, we selected the period from 1 January 1958 to 31 December 1979, which is considered representative of the climate conditions over the preceding few hundred years,^46^^,^^47^ as the repeated baseline reference climate interval (RCI) time series. Equilibrium is roughly reached when the entire firn column (defined here as the depth at which the density reaches the pore close-off density of 830 kg m^−3^) has been fully refreshed by accumulation.^17^ Due to the variability in snow accumulation rates, the required spin-up time can vary significantly, resulting in a self-adaptive time frame where the 22-year climate interval is repeated iteratively to generate a time series sufficiently long to ensure full refreshment of the firn column.

### Method details

Transformer networks have demonstrated superior performance in tasks such as natural language processing, S2S conversion, and time series prediction.^48^^,^^49^^,^^50^ In firn density prediction, the primary challenge lies in the limited availability of *in situ* firn density data and the limited knowledge of the complex densification mechanisms. Firn deposition is a continuous, time-dependent process, where the depth-density profile reflects layer-specific responses to deposition conditions and the firn stratigraphy exhibits long-distance interrelationships. The transformer is particularly effective in handling problems with complex mappings where accurate physical models are either unavailable or challenging to construct, as it can capture both global features and local relationships layer by layer. However, directly applying transformer networks for firn density prediction may face inherent limitations, as purely data-driven approaches struggle to achieve robust performance given the sparse nature of *in situ* firn density observations. Therefore, we propose the FDTransformer, which combines the data-driven characteristics of a sequence self-attention transformer network with the physical properties of firn densification to simulate firn density profiles. On the one hand, the transformer network is used to fully explore the complex mapping relationships between eight firn parameters and firn density, thus reducing the dependency of densification models on idealized assumptions and the uncertainties arising from limited knowledge of densification mechanisms. On the other hand, eight physically constrained parameter sequences derived from the physics-based densification model serve as input sequences to the FDTransformer, effectively integrating physical priors into the data-driven framework. The framework of the proposed FDTransformer model for firn density estimation is shown in Figure 4.

#### Architecture of the FDTransformer

The proposed FDTransformer model architecture for firn density prediction is illustrated in Figure 5. The model transforms input sequences of physical parameters (POR, AGE, GS, COM, AR, OS, T, and M) into output density sequences (DEN-pred) of equal length. Each input and target density (DEN-true) sequence spans 4.5 meters, comprising 450 data points where each point represents the property value at a specific depth.

The model begins with an input embedding block that maps the physical parameter sequences into a high-dimensional feature space. This block consists of a convolution block followed by four residual convolution blocks. The convolution block (Figure S4A) is designed to extract localized short-term features while modeling inter-point dependencies within sequential data. In the convolutional block, the feature dimension is set to 64, a value commonly used as an intermediate representation size in 1D convolutional tasks. Each residual convolution module (Figure S4B) enhances feature learning through skip connections that add the input directly to the output after two consecutive convolution operations.^51^ This residual connection helps the model retain more information and promotes deeper network training. Subsequently, a positional encoding block is employed to map the depth information to a fixed-sized vector, which is then element-wise added to the output of the input embedding block, providing the model with positional information about data points in the depth direction.

The model consists of 4 identical transformer encoder layers, each containing two key submodules. First, a multi-head attention module employs 4 parallel attention heads to capture complex relationships across different positions in the sequence (Figure S4C). Each attention head independently computes scaled dot-product attention in a reduced-dimensional subspace, allowing the model to learn diverse representations. The outputs from all attention heads are concatenated and linearly projected to maintain dimensional consistency with the input feature space. Second, a position-wise feed-forward network with a hidden layer dimension of 256 applies two linear transformations with GELU activation to further refine the features. Both submodules incorporate residual connections and layer normalization to stabilize training and facilitate gradient flow.

The model comprises a decoder layer, which incorporates 4 consecutive residual convolution blocks. These blocks progressively transform the features while maintaining robustness through their residual connections. Finally, the decoder uses a 1D convolution layer followed by a sigmoid activation function to map the high-dimensional features to the predicted density values, enabling direct comparison with the target density curves.

During network training and validation, we optimize the model using the mean absolute error (L1 loss) between the predicted density curve and the corresponding target density curve. To enhance the model’s performance in predicting density curves, several advanced optimization strategies are implemented during training and validation. First, the Adam optimizer^52^ is used to update the parameters of the transformer network, chosen for its efficiency in handling sparse gradients and its adaptive learning rate capabilities. Second, dropout layers are incorporated into the transformer encoder to prevent overfitting, with a dropout rate of 0.1 applied across all layers. Third, an adaptive learning rate decay strategy is implemented to accelerate convergence and further reduce the risk of overfitting, with an initial learning rate of 10^-4^ and a minimum rate of 10^-7^. The learning rate decreases as the number of epochs increases. Specifically, the learning rate is multiplied by 0.2 (i.e., reduced to 20% of its current value) if the validation loss does not improve over 30 consecutive epochs. Fourth, an early stopping strategy^53^ is applied to stop model training and save the optimal parameters if no improvement in validation loss is observed for 150 consecutive epochs within a maximum of 1000 epochs, thereby preventing overfitting and ensuring efficient training. The model’s hyperparameters mentioned above are carefully determined through a systematic optimization process involving both grid search and manual tuning based on validation set performance, ensuring optimal model effectiveness with efficient training.

#### Data pre-processing

We collect 8, 10, and 5 density profiles at Dye-2, KAN_U, and Summit, respectively, for training, validation, and testing. At each site, a profile is selected as the test profile for model evaluation. The remaining profiles are used to generate the training and validation sets, serving for model training and model parameters selection, respectively. To ensure that all features contribute equally to the model and to prevent features with larger ranges from dominating the training process, Min-Max normalization is applied to rescale the eight parameter curves and the target density curve to the range [0, 1]. This normalization follows the formula:

(Equation 1) $$X_{n}=\frac{X-X_{\min}}{X_{\max}-X_{\min}}\text{,}$$

Where *X*_n_ denotes the normalized curves, *X* denotes the original curves, *X*_max_ and *X*_min_ represent the maximum and minimum values of the corresponding curve, respectively. It should be noted that the maximum and minimum values used in this study are the global maximum and minimum values across all profiles at each site.

#### Training and validation

After resampling and normalization, each curve is divided into segments of 5 m (corresponding to 500 samples) from top to bottom (Figure 6A). The length of 5 m is chosen based on its ability to adequately capture data dependencies while maintaining computational efficiency (as explained in discussion). If the last segment contains fewer than 500 samples, zero padding is applied to maintain uniformity. Subsequently, we apply a K-fold cross-validation technique, which helps enhance generalization and adequately utilize the available data, to split the segments of training profiles into training and validation sets. The training profiles are divided into K subsets, where K equals the number of training profiles at each site, meaning each subset contains segments from one profile. In each fold, K-1 subsets are used for model training, and the remaining subset is used for model validation (i.e., model parameters selection). Each subset is used for validation exactly once, ensuring that every data point contributes to both training and validation. To further enhance the robustness of model evaluation, we repeat the K-fold cross-validation process 20 times.

Data augmentation is an effective strategy for enhancing model generalization capability in few-shot learning scenarios.^54^ However, conventional image augmentation strategies such as rotation, flipping, and adding noise are not suitable for firn density estimation, as these techniques would alter the original geological meaning of firn parameter curves.^55^ Therefore, we apply a data augmentation strategy based on random starting point cropping to maximize the utilization of available density data. As shown in Figure 6B, after each curve is segmented, a 5m-long sequence is available. For each sequence, only 4.5m-long data are used during training and validation, resulting in a redundant length of 0.5 m (yellow rectangular box). The random starting point cropping strategy randomly selects a starting point within the first 0.5 m of the sequence and extends 4.5 m downward (blue line interval) to form the training and validation sequences. This approach ensures that the training and validation sequences are slightly different for each epoch, effectively expanding the training dataset size and improving generalization capability.

#### Firn density prediction

After the FDTransformer model has been trained, the test profile is segmented into 4.5m-long sequences. The FDTransformer model uses the optimal model parameters from each fold to process these sequences and generate the corresponding density sequences (DEN-pred). To maintain data continuity when reconstructing the complete density profile from the sequences from each fold, we introduce overlap between adjacent sequences, which minimizes boundary effects and allows for a smooth transition between sequences. Subsequently, we employed a weighted averaging approach to obtain the predicted density profile for this round of cross-validation. Specifically, the weight for each fold is determined based on the mean absolute error (MAE) between the true density and the predicted density obtained using the optimal weights from the validation sequences, with lower MAE values corresponding to higher weights. This weighted averaging ensures that more accurate predictions from individual folds contribute more heavily to the final output. The predicted density profiles from the 20 cross-validation runs are averaged and then inverse normalized to return the predictions to their original scale, allowing for direct comparison with the actual measured density profiles.

### Quantification and statistical analysis

Several common regression evaluation metrics, including the correlation coefficient (R), Nash-Sutcliffe efficiency coefficient (NSE), normalized root mean square error (NRMSE), and MAE, are used for statistical analyses. All statistical analyses are performed using Python 3.10 (Numpy 1.26.4 and matplotlib 3.7.0 packages). R quantifies the relationship between observations and simulations. NSE is commonly used to assess the goodness of model simulations, particularly in hydrological modelling.^56^ NSE ranges from negative infinity to 1, where values closer to 1 indicate superior simulation accuracy. An NSE value of 1 represents perfect model performance, and a value of 0 suggests that the simulated results align with the mean level of the observed values. It is calculated as:

(Equation 2) $$\text{NSE}=1-\frac{\sum_{i=1}^{n}{\left(O_{i}-P_{i}\right)}^{2}}{\sum_{i=1}^{n}{\left(O_{i}-\bar{O}\right)}^{2}}\text{,}$$

where *P*_*i*_ is the predicted density, *O*_*i*_ is the observed density, $\bar{O}$ is the average value of the observed density. NRMSE and MAE are widely used to assess the accuracy of model simulations relative to observations. NRMSE is calculated as the root mean square error divided by the standard deviation of observations, enabling comparability across datasets with varying dispersion. NRMSE quantifies the relative magnitude of model simulation errors, where values approaching 0 indicate nearly perfect agreement between simulations and observations, values below 1 represent errors within the range of observational variability, and values at or above 1 suggest substantial deviations where simulation errors exceed the inherent density variability. MAE provides an intuitive and robust measure of average error magnitude without sensitivity to extreme values. Lower MAE values indicate better agreement between simulations and observations, implying higher accuracy of the model. The formulas for NRMSE and MAE are:

(Equation 3) $$\text{NRMSE}=\frac{\sqrt{\frac{\sum_{i=1}^{n}{\left(P_{i}-O_{i}\right)}^{2}}{n}}}{\sqrt{\frac{\sum_{i=1}^{n}{\left(P_{i}-\bar{O}\right)}^{2}}{n}}}\text{,}$$

(Equation 4) $$\text{MAE}=\frac{1}{n}\sum_{i=1}^{n}\left|O_{i}-P_{i}\right|\text{,}$$

where *n* denotes the number of depth points within a specific depth range.
