# Supplementary material for: FDTransformer: A firn density prediction framework combining a self-attention transformer network with firn densification physics
Source: iScience. 2025 Oct 28;28(11):113869. doi: 10.1016/j.isci.2025.113869 (PMC12661306; doi:10.1016/j.isci.2025.113869)

iScience, Volume *28*

Supplemental information

FDTransformer: A firn density prediction framework combining a self-attention transformer network with firn densification physics

Xueyu Zhang, Lin Liu, Houjun Jiang, and Zhicai Luo

Figure S1. Each measured depth-density profile collected at Dye-2

Profile D3 is selected as the test profile and the remaining profiles are used as the training profiles.

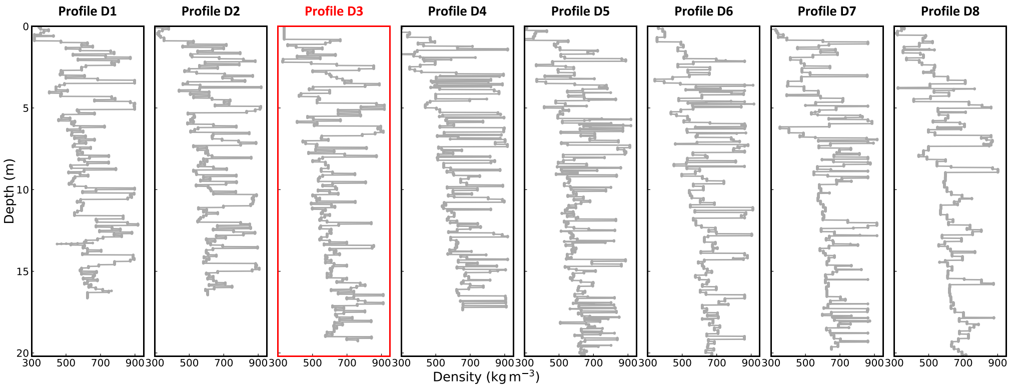

Figure S2. Each measured depth-density profile collected at KAN_U

Profile K8 is selected as the test profile and the remaining profiles are used as the training profiles.

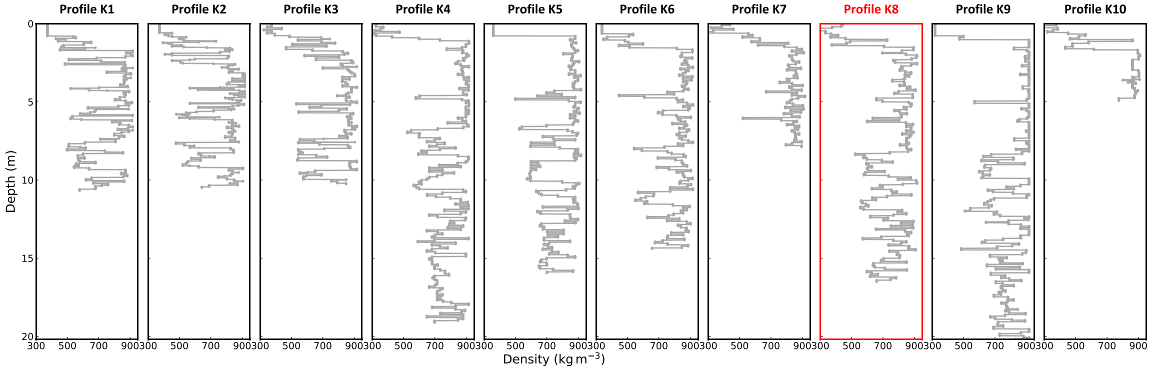

Figure S3. Each measured depth-density profile collected at Summit

Profile S4 is selected as the test profile and the remaining profiles are used as the training profiles.

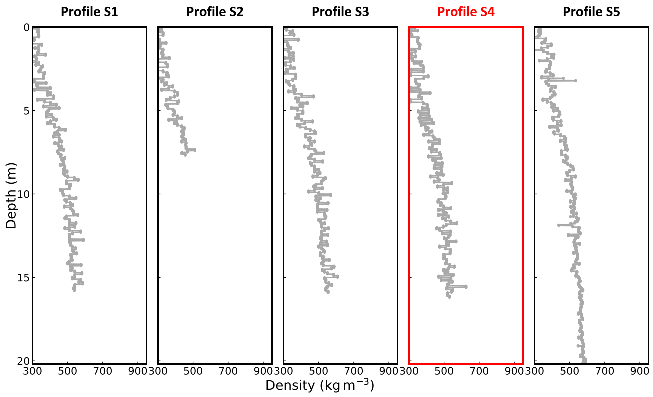

Figure S4. A detailed diagram of four main modules in the FDTransformer architecture
(a) the composition of a convolution block.

(b) the composition of a residual convolution block.

(c) the composition of a multi-head attention module.

(d) the composition of a feed forward layer.

The Conv1d represents the 1D convolution layer with 64 kernels, a kernel size of 11, padding of 5, and a stride of 1. SA represents the self-attention module. ReLU and GELU are the rectified linear unit activation function and the gated rectified linear unit activation function, respectively.

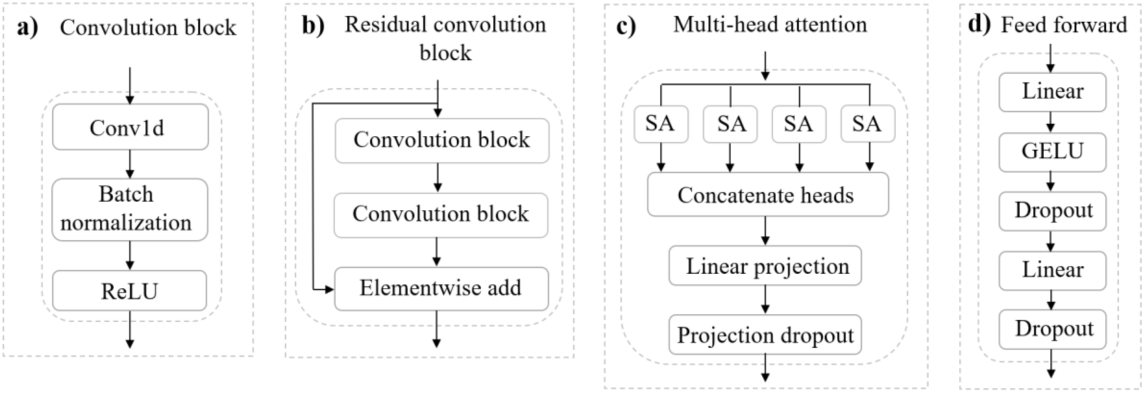

Supplement: Document S1. Figures S1–S4 [file mmc1.pdf]
